# Clinical Factors Associated with Brain Volume Reduction in Systemic Lupus Erythematosus Patients without Major Neuropsychiatric Manifestations

**DOI:** 10.3389/fpsyt.2018.00008

**Published:** 2018-02-01

**Authors:** Shuang Liu, Yuqi Cheng, Yueyin Zhao, Hongjun Yu, Aiyun Lai, Zhaoping Lv, Xiufeng Xu, Chunrong Luo, Baoci Shan, Lin Xu, Jian Xu

**Affiliations:** ^1^Department of Rheumatology and Immunology, First Affiliated Hospital of Kunming Medical University, Kunming, China; ^2^Department of Psychiatry, First Affiliated Hospital of Kunming Medical University, Kunming, China; ^3^Magnetic Resonance Imaging Center, The First Hospital of Kunming, Kunming, China; ^4^Key Laboratory of Nuclear Analysis, Institute of High Energy Physics, Chinese Academy of Sciences, Beijing, China; ^5^Key Laboratory of Animal Models and Human Disease Mechanisms, Kunming Institute of Zoology, Chinese Academy of Sciences, Kunming, China

**Keywords:** systemic lupus erythematosus, quantitative magnetic resonance imaging, brain volume reduction, cognitive impairment, immunosuppressive agents

## Abstract

The aim of the study was to find structural brain changes in systemic lupus erythematosus patients without major neuropsychiatric manifestations [non-neuropsychiatric systemic lupus erythematosus (non-NPSLE)] using quantitative magnetic resonance imaging (MRI) and possible associations with clinical characteristics. 89 non-NPSLE patients with normal conventional MRI and 84 healthy controls (HCs) were recruited. The whole brain gray matter volume (GMV) and white matter volume (WMV) were calculated for each individual. We found obvious GMV and WMV reduction in the systemic lupus erythematosus (SLE) group compared with HCs. Female patients showed significant reduction of GMV and WMV compared with male patients. Patients treated with immunosuppressive agents (ISA) showed less WMV reduction than those without. Cognitive impairment was the most common subclinical neuropsychiatric manifestation and had a prevalence of 46.1%. Association between WMV reduction with cognitive impairment was found. Thus, we concluded that structural brain atrophy could happen even before occurrence of obvious neuropsychiatric signs and symptoms and was associated with subclinical symptoms such as cognitive impairment. ISA treatment might have a protective effect on the brain atrophy. Early treatment might prevent the progressive damage to the brain. More studies are needed to fully understand the complicated underlying mechanisms of brain atrophy in SLE.

## Introduction

Systemic lupus erythematosus (SLE) is an autoimmune disease involving almost all organ systems. Central nervous system (CNS) involvement is typical during the course of SLE (1, 2). Brain atrophy, which has long been reported in SLE using neuroimaging techniques, often correlates with clinical manifestations, even in patients without obvious CNS signs and symptoms (3, 4). Magnetic resonance imaging (MRI) is widely used to detect brain anatomical abnormalities in SLE patients, including cerebral atrophy (3, 5, 6). However, conventional or anatomical MRI findings are sometimes non-specific and can be negative in neuropsychiatric SLE (NPSLE) patients (7). Many patients with only mood or cognitive disorders have been identified as normal according to conventional MRI. There is evidence that abnormal white matter (WM) structures were found by MRI volumetrics in SLE patients without major neuropsychiatric manifestations, i.e., non-NPSLE patients and SLE patients with normal conventional brain MRI, suggesting that structural abnormalities could happen before obvious CNS manifestations (8). If subclinical brain structure involvement could be identified before the emergence of obvious neuropsychiatric symptoms, earlier intervention could be initiated, potentially preventing progression of brain injury.

Here, we tried to evaluate the brain structural abnormalities with quantitative MRI in a relatively large sample of non-NPSLE patients with normal conventional MRI and explore the potential associations between the clinical factors and these possible brain abnormalities.

## Materials and Methods

### Subjects

Systemic lupus erythematosus patients in the inpatient and outpatient divisions of the Rheumatology and Immunology Department of First Affiliated Hospital of Kunming Medical University, which is a member unit of Chinese SLE Treatment and Research Group, were recruited in this study from September 2012 to September 2014. All participants were studied following a standardized protocol and evaluated by the same investigator throughout the course of the study. Before enrollment into the study, each participant provided a written informed consent after receiving a complete description of the study. This research was approved by the Institutional Review Board of Kunming Medical University, Yunnan Province, PR China (ClinicalTrials.gov: NCT00703742).

The inclusion criteria were as follows: (1) patients diagnosed as SLE according to the 1997 revised American College of Rheumatology (ACR) criteria for the classification of SLE (9); (2) patients between the ages of 16 and 50 years; and (3) patients willing to attend this study and give written informed consents.

The exclusion criteria were as follows: (1) patients fitting the ACR diagnostic criteria for rheumatoid arthritis, systemic sclerosis, Sjögren’s syndrome (primary or secondary) or other connective tissue diseases, and drug-induced SLE; (2) patients with organic brain or neurological disorders that would disturb the structure or diffusion imaging of the brain (i.e., history of head trauma, Parkinson’s disease, or seizures); (3) patients with major active psychiatric manifestations, such as obvious disorganized behaviors and conscious disturbances; (4) patients with history of substance abuse; (5) patients who were pregnant or suspected to be pregnant; (6) patients with contraindication to MRI, such as claustrophobia or cardiac pacemakers; (7) patients with serious clinical conditions that could cause cerebral atrophy, such as history of arterial hypertension, diabetes mellitus, stroke, or renal insufficiency; and (8) patients with structural abnormities of the brain identified by conventional T1- and T2-weighted MRI.

98 SLE patients were included. All 98 patients received full sets of laboratory tests, disease activity evaluations, questionnaires, and MRI scans. 95 healthy controls (HCs) with age and sex matched were recruited. A complete general physical examination, including neurological examinations, was applied to each HC by a rheumatologist and a neurologist in order to exclude major disorders especially neurological diseases. Psychiatric symptoms were screened by a psychiatrist using the Structured Clinical Interview for the Diagnostic and Statistical Manual of Mental Disorders-IV Non-Patient Version. All participants were Chinese Han people and were right-handed.

### Clinical Characteristics of SLE Patients

Data such as gender, age, and disease duration were collected for each patient. Disease duration was defined as the period from the initial manifestations of SLE to the day of MRI acquisition. All clinical manifestations and laboratory test findings were recorded. Autoantibodies including antinuclear antibody, anti-double stranded DNA antibody, anti-Sm antibody, anti-nucleosome antibody, anti-U1 ribonucleoprotein antibody, anti-histone antibody, anti-ribosomal P0 antibody, anti-SSA 52 kDa antibody, anti-SSA 60 kDa antibody, anti-SSB antibody, anti-cardiolipin (aCL) antibody, and lupus anticoagulant were all recorded. Disease activity was measured by the SLE disease activity index (SLEDAI), and cumulative SLE-related damage was determined by the Systemic Lupus International Collaborating Clinics/American College of Rheumatology Damage Index for Systemic Lupus Erythematosus in all SLE patients at the same day of the MRI. Active disease status was defined as a SLEDAI score of higher than 9 (10–12).

The dosages of glucocorticoids and immunosuppressive agents (ISA) used from the initiation of treatment till the day of MRI were collected by careful interviews. The cumulative dosages were calculated by adding up the daily dosages. The total dosages of oral and intravenous glucocorticoids were converted to equivalent dosages of prednisone.

A complete neurological examination was applied to all subjects in order to exclude major neurological diseases, such as stroke and seizures. Subjects with obvious disorganized behaviors and psychiatric symptoms, such as illusion and delusion, were also excluded. All participants were right-handed, as assessed by the Edinburgh Handedness Inventory (13). All clinical data were collected on the MRI examination day by an experienced psychiatrist. The cognitive status was evaluated by mini-mental state examination (MMSE). Cognitive impairment was defined when patients had total scores less than 27. The depression status was evaluated by Hamilton Depression Scale (HAMD), and anxiety was evaluated by Hamilton Anxiety Scale (HAMA). Patients were diagnosed as depression when the total scores of HAMD greater or equal to 17 and anxiety with HAMA greater or equal to 14.

### MRI Images Acquisition

Magnetic resonance imaging images acquisition was performed by an experienced neuroradiologist. MRI sequences were performed on all subjects with a 1.5T clinical MRI scanner manufactured by General Electric Company (Twin speed; Milwaukee, WI, USA) equipped with a birdcage head coil. Supportive foam pads were used to minimize head motion. A rapid sagittal localizer scan was acquired to confirm alignment. Normal T1 and T2 MRI scans were taken to exclude obvious structural abnormalities. In the 98 SLE patients, nine patients were excluded due to brain structural abnormities identified by T1- and T2-weighted MRI (local infarction or ischemia). Data from the remaining 89 patients were included in this study. 11 subjects from the HC group were also excluded due to local ischemia, and thus 84 HC subjects were included in the study. A set of three-dimensional volumetric structural MRI scans was taken on each subject using a fast spoiled gradient echo sequence with the following parameters: repetition time/echo time = 10.5/2 ms, matrix size = 256 × 256, thickness = 1.8 mm with no interslice gap, field of view = 240 mm, and flip angle = 15°. Resolution = 0.94 mm× 0.94 mm× 0.9 mm. Whole brain images were acquired in axial planes parallel to the anterior commissure-posterior commissure line, yielding 172 continuous slices of 0.9 mm thick.

### Data Preprocessing and Voxel-Based Morphometry (VBM) Statistical Analysis

DICOM image data were processed *via* MRIcro software (version 1.40^1^). All data were analyzed *via* statistical parametric mapping (SPM) 5 (Wellcome Department of Cognitive Neurology, London, UK^2^) and VBM 5^3^ software based on Matlab 7.1 (The MathWorks, Inc., Natick, MA, USA). Each individual image was normalized and transformed into the standardized Montreal Neurological Institute template, and then resampled at the 2 mm × 2 mm × 2 mm dimensional scale. Normalized images were then segmented into gray matter (GM), WM, and cerebrospinal fluid (CSF). Modulated GM and WM images were then smoothed to remove noise using a filter with a half-width half maximum of 8 mm.

### Analysis of Whole Brain Gray Matter Volume (GMV) and White Matter Volume (WMV)

Initially, we used the standard GM and WM templates implanted in SPM 5 as the whole brain GM and WM masks. Then, using the smoothed modulated GM and WM images from each participant, the mean GMV and WMV were retrieved. Covariance analyses with age and total brain volume as covariant factors were then performed to analyze the differences in GMV and WMV between SLE and HC groups as well as different subgroups of SLE patients with SPSS 17.0 (SPSS Inc., 1989–2004). We used Bonferroni methods for correction in multiple comparisons. Partial correlation analyses with age and total brain volume controlled were used to explore the possible correlations between disease characteristics and the GMV and WMV. The results were statistically significant when *p* < 0.05. All statistical tests were two-sided.

## Results

### Demographic Data of SLE and HC Groups

89 SLE and 84 HC were analyzed in this study. The mean age was 28.69 years (SD = 7.40, range 16–48) in SLE patients and 30.35 years (SD = 7.80, range 17–50) in the HC group. There were no significant differences in age or gender between these two groups. Detailed data are shown in Table 1.

**Table 1:** Demographic and clinical characteristics of SLE and HC groups.

	SLE (*n* = 89)	HC (*n* = 84)	t	p
Age (years, mean ± SD)	28.69 ± 7.40	30.35 ± 7.80	−1.795	0.074
Female/male	74/15	64/20	1.295 (χ^2^)	0.255
Disease duration (m, mean ± SD)	19.39 ± 28.36	NA		
SLEDAI (mean ± SD)	9.71 ± 6.20	NA		
Total steroids (g, mean ± SD)	9.27 ± 13.25 (*n* = 70)	NA		
Total CTX (g, mean ± SD)	4.15 ± 3.41 (*n* = 23)	NA		
Total HCQ (g, mean ± SD)	54.36 ± 94.40 (*n* = 37)	NA		

*SLE, systemic lupus erythematosus; HC, healthy control; NA, not applicable; SLEDAI, SLE disease activity index; CTX, cyclophosphamide; HCQ, hydroxychloroquine*.

### Clinical Characteristics of SLE Patients

The disease duration in SLE patients ranged from 0.25 to 204 months (mean = 19.39 months, SD = 28.36). 41 patients were newly diagnosed with SLE in our department, and we defined them as new onset SLE. While the other 48 patients with previous definite diagnosis of SLE were defined as established SLE. At the time of MRI scanning, the mean SLEDAI score was 9.71 (SD = 6.20), and 49 patients were defined as active. In the 89 patients, 54 received ISA [17 with cyclophosphamide (CTX), 31 with hydroxychloroquine, and the other six with both]. Detailed data are shown in Table 1.

According to the cognitive and emotional scales, 41 (46.1%) patients were with cognitive impairment, 12 (13.5%) patients and 13 (14.6%) patients were in depression and anxiety status, respectively.

### GMV and WMV Differences between SLE and HC Groups

The GMV and WMV were compared between SLE patients and HC groups. Both the GMV and WMV were significantly decreased in the SLE group, as compared with the HC group. Detailed data are shown in Table 2.

**Table 2:** GMV and WMV differences between different subgroups.

	GMV		*p*	WMV		*p*
	Mean	SD		Mean	SD	
SLE (*n* = 89)	0.3965	0.0331	<0.001**	0.3762	0.0377	0.001**
HC (*n* = 84)	0.4251	0.0355		0.3971	0.0392	
Treated (*n* = 54)	0.3995	0.0327	0.339	0.3831	0.0354	0.039*
Untreated (*n* = 35)	0.3918	0.0338		0.3662	0.0393	
Active (*n* = 49)	0.3952	0.0336	0.673	0.3782	0.0398	0.563
Inactive (*n* = 40)	0.3978	0.0331		0.3742	0.0359	
New onset (*n* = 41)	0.3928	0.0334	0.407	0.3722	0.0384	0.371
Established (*n* = 48)	0.3996	0.0330		0.3797	0.0372	
Male (*n* = 15)	0.4214	0.0346	0.002**	0.4121	0.0306	<0.001**
Female (*n* = 74)	0.3914	0.0307		0.3690	0.0349	
MMSE pos (*n* = 41)	0.3910	0.0315	0.344	0.3667	0.0375	0.029*
MMSE neg (*n* = 48)	0.4011	0.0342		0.3844	0.0364	
HAMD pos (*n* = 12)	0.3867	0.0260	0.318	0.3677	0.0297	0.413
HAMD neg (*n* = 77)	0.3980	0.0340		0.3776	0.0388	
HAMA pos (*n* = 13)	0.3845	0.0242	0.274	0.3649	0.0305	0.257
HAMA neg (*n* = 76)	0.3985	0.0342		0.3782	0.0387	

*GMV, whole brain gray matter volume; WMV, whole brain white matter volume; SLE, systemic lupus erythematosus; HC, healthy control; MMSE, mini-mental state examination; pos: positive; neg: negative; HAMD, Hamilton Depression Scale; HAMA, Hamilton Anxiety Scale*.

*p < 0.05.

**p < 0.01.

### Association between GMV/WMV and Clinical Characters

We found gender differences in the GMV and WMV. Female patients showed more significant reduction of GMV and WMV than male patients (0.3914 ± 0.0307 vs. 0.4214 ± 0.0346; *p* = 0.002; 0.3690 ± 0.0349 vs. 0.4121 ± 0.0306; *p* < 0.001; respectively). Patients treated with ISA had higher WMV than those untreated (0.3831 ± 0.0354 vs. 0.3662 ± 0.0393; p = 0.039), while no difference was found in GMV. We found no significant differences of GMV and WMV between active and inactive or between new onset and established SLE patients. According to the cognitive and emotional scales, patients were divided into two groups, dysfunctional status as positive (shortly as pos) groups and normal patients as negative (shortly as neg) groups. Then, we found that patients with cognitive dysfunction showed more WMV reduction than patients with normal cognitive status (*p* = 0.029). We found no significant differences between positive and negative groups of all the autoantibodies. Detailed data are shown in Table 2.

We found a negative correlation between age and GMV (*r* = −0.295, *p* = 0.005). The cumulative steroid dosage was positively correlated with GMV and WMV (*r* = 0.227, *p* = 0.033; *r* = 0.250, *p* = 0.019, respectively). We also found a tendency of positive correlation between MMSE score and WMV (*r* = 0.210, *p* = 0.050). Detailed data are shown in Table 3.

**Table 3:** Correlation between GMV/WMV and clinical characters.

	GMV		WMV	
	*r*	*p*	*r*	*p*
Age	−0.295	0.005*	−0.030	0.777
SLEDAI	−0.042	0.701	0.059	0.586
Disease duration (m)	0.125	0.267	093	0.407
Total steroids (g)	0.227	0.033*	0.250	0.019*
Total HCQ (g)	0.137	0.204	0.108	0.317
Total CTX (g)	0.093	0.388	0.189	0.078
MMSE	0.099	0.360	0.210	0.050
HAMD	−0.187	0.081	−0.142	0.187
HAMA	−0.151	0.161	−0.159	0.138

*GMV, whole brain gray matter volume; WMV, whole brain white matter volume; SLEDAI, systemic lupus erythematosus disease activity index; HCQ, hydroxychloroquine; CTX, cyclophosphamide; MMSE, mini-mental state examination; HAMD, Hamilton Depression Scale; HAMA, Hamilton Anxiety Scale*.

*p < 0.05.

## Discussion

In this study, we found significant GMV and WMV reductions in non-NPSLE patients who had normal conventional MRI with significant gender differences. Although MRI is considered as a useful method for evaluations of CNS manifestations in SLE patients, conventional or anatomical MRI findings are often non-specific or negative in patients with or without CNS manifestations (7). The patients included in this study had normal conventional MRI and no major CNS manifestations, so the GM and WM loss implied that brain atrophy could occur even before obvious clinical neuropsychiatric manifestations. Consistent with a previous magnetic resonance spectroscopy (MRS) study, our results confirmed that abnormal structural brain changes could occur in SLE patients without obvious CNS symptoms or abnormal conventional MRI findings, whom we considered as non-NPSLE patients before. We found an even higher prevalence of cognitive impairment than the 25% prevalence in the MRS study (8). Thus, the method we used could calculate the accurate quantitative whole brain GMV and WMV and detect structural GM and WM reductions of SLE patients, which might aid in predicting NPSLE and evaluating the cumulative damages in SLE patients.

We found a gender difference in our study. Female SLE patients had significant lower whole brain GMV and WMV than male patients. It was consistent with another study of our team, in which we found that both sides of brain hippocampal volume were significantly decreased in women compared to men (unpublished result). However, we did not find similar results in other SLE-related research studies. We hypothesized that these findings might be related to the higher estrogen levels in female, which were also considered as one of the reasons of the higher prevalence of SLE in females. Some researchers believed that gender differences could be found in almost all brain activities (14).

We found that cognitive impairment was quite common in non-NPSLE patients with a prevalence of about 46.1% and MMSE score had a tendency of positive correlation with WMV. Several research studies have shown that cognitive dysfunction was one of the most common and underestimated neuropsychiatric manifestations, and there were associations between cognitive dysfunction and brain atrophy (15–17). Certain antibodies such as aCL antibodies were reported to contribute to the cognitive impairment through activation of inflammation (18). The associations between depression or depressive syndromes and diffuse or local brain atrophy were reported in idiopathic depression, elderly people, Alzheimer’s disease, and other diseases, however, were not much reported in SLE (19–23). Our study also showed no significant associations between depression and brain atrophy in SLE. More studies are needed to reveal the situation better.

This study also revealed that patients treated with ISA had increased WMV compared with those untreated. This finding suggested a potential protective role of immunosuppressive treatment in preventing brain atrophy, which is consistent with our previous finding (24). The potential neuroprotective effects of CTX have been confirmed in SLE and other CNS diseases such as antiphospholipid syndrome and experimental autoimmune GM disease in animal experiments (25–28). However, for glucocorticoids, we found a positive correlation between cumulative glucocorticoids dosage and GMV/WMV, which was not found in our previous study (24). Although we used glucocorticoids in the treatment of SLE including NPSLE, several researchers reported the association or positive correlation of glucocorticoids and brain atrophy or brain lesions, which showed the complex effects of glucocorticoids on CNS in SLE patients (15, 17, 28–31). More research studies are needed to reveal the exact effects of glucocorticoids in SLE. Thus, early immunosuppressive treatments might prevent the brain atrophy.

The brain atrophy has been previously reported in SLE (5, 32, 33). However, the exact mechanism remains unclear. Several factors have been reported to contribute to it. Autoantibodies, which played a key role in the pathogenesis of SLE, were also involved in the pathogenesis of CNS damage. Typical antibodies, such as aCL antibodies and anti-ribosomal P0 antibodies, were widely reported in related research studies and could affect both small vascular and brain parenchyma. Other antibodies, such as anti-Sm antibodies and anti-SSB antibodies, were also reported by some researchers (17, 18, 30, 34–38). However, in our study we found no significant associations between autoantibodies and brain volume reduction. The dysfunction of blood–brain barrier (BBB) was also believed to participate in the CNS disorders. The BBB was the main barrier preventing systemic antigens from circulating to the CNS, and it could be damaged by certain pathways mediated by immunoreaction (39). Thus, systemic and local cytokine productions could be initiated and damage the CNS together with possible vasculopathy in the brain, as researchers have reported elevation of tumor necrosis factor-α, interleukin-6 (IL-6), and IL-10 in CSF in SLE patients (40). The immune-mediated inflammation could thus cause further damage to neurons, axons, and BBB backwards. Then, clinical or subclinical neuropsychiatric symptoms occurred (7). More research studies exploring the mechanisms are needed for a full understanding.

There are still some flaws in our study. One is that we used SPM and VBM software to reflect the group difference. Because it was reported that without prior probability maps, segmentation with SIENAX software might result with GM underestimation and CSF overestimation (41). Thus, in the unified segmentation model of VBM, we used warping to match individuals’ scans and modulation to obtain the volume. Although there might be a possibility of increasing the bias of the data, we considered our method could be a useful tool to evaluate the brain volume in SLE patients.

In this study, we used a non-invasive quantitative MRI technique for early detection of structural brain atrophy, which was associated with subclinical neuropsychiatric manifestations in SLE patients. Because we chose non-NPSLE patients without obvious neuropsychiatric symptoms or abnormal conventional MRI, the results suggested that the brain involvement was a primary deficit in SLE. Immunosuppressive therapy had early neuroprotective effects and might prevent progressive brain damage. More studies are needed to find the underlying complex mechanisms of CNS disorders in SLE.

## Ethics Statement

This study was carried out in accordance with the recommendations of the clinical trial guidelines of the Institutional Review Board of Kunming Medical University with written informed consent from all subjects. All subjects gave written informed consent in accordance with the Declaration of Helsinki. The protocol was approved by the Institutional Review Board of Kunming Medical University.

## Author Contributions

SL, YC, and YZ were responsible for the management of the research and writing the article. AL and ZL were responsible for recruiting and following up the patients. HY and CL were responsible for doing MRI. XX, BS, and LX were responsible for the consultation of the research. JX was responsible for the whole research and article.

## Conflict of Interest Statement

The authors declare that the research was conducted in the absence of any commercial or financial relationships that could be construed as a potential conflict of interest.

## Footnotes

^1^http://www.mricro.com.

^2^http://www.fil.ion.ucl.ac.uk/.

^3^http://dbm.neuro.uni-jena.de/vbm/vbm5-for-spm5/.
